# Effect of fusion protein cleavage site sequence on generation of a genotype VII Newcastle disease virus vaccine

**DOI:** 10.1371/journal.pone.0197253

**Published:** 2018-05-14

**Authors:** Vinoth K. Manoharan, Berin P. Varghese, Anandan Paldurai, Siba K. Samal

**Affiliations:** Virginia-Maryland Regional College of Veterinary Medicine, University of Maryland, College Park, Maryland, United States of America; St. Jude Children's Research Hospital, UNITED STATES

## Abstract

Newcastle disease (ND) causes severe economic loss to poultry industry worldwide. Frequent outbreaks of ND in commercial chickens vaccinated with live vaccines suggest a need to develop improved vaccines that are genetically matched against circulating Newcastle disease virus (NDV) strains. In this study, the fusion protein cleavage site (FPCS) sequence of NDV strain Banjarmasin/010 (Banj), a genotype VII NDV, was individually modified using primer mutagenesis to those of avian paramyxovirus (APMV) serotypes 2, 7 and 8 and compared with the recombinant Banjarmasin (rBanj) with avirulent NDV LaSota cleavage site (rBanj-LaSota). These FPCS mutations changed the *in vitro* cell-to-cell fusion activity and made rBanj FPCS mutant viruses highly attenuated in chickens. When chickens immunized with the rBanj FPCS mutant viruses and challenged with the virulent Banj, there was reduced challenge virus shedding observed compared to chickens immunized with the heterologous vaccine strain LaSota. Among the genotype VII NDV Banj vaccine candidates, rBanj-LaSota and rBanj containing FPCS of APMV-8 induced highest neutralizing antibody titers and protected chickens with reduced challenge virus shedding. These results show the effect of the F protein cleavage site sequence in generating genotype VII matched NDV vaccines.

## Introduction

Newcastle disease virus (NDV) causes a severe disease in chickens worldwide. NDV is an enveloped virus belonging to family *Paramyxoviridae*. NDV has a negative-sense, nonsegmented RNA genome that contains six genes (3’-N-P-M-F-HN-L-5’) [1,2]. NDV contains two envelope glycoproteins, the hemagglutinin-neuraminidase (HN) protein and the fusion (F) protein. The HN protein is responsible for binding to cell surface receptor and the F protein is responsible for fusion between the viral envelope and the cell membrane [2,3]. The F protein is synthesized as an inactive F_0_ precursor, which must be cleaved by host cell proteases into F_1_ and F_2_ subunits [4]. The cleavage of F protein is the major determinant of NDV virulence [2–5]. The avirulent NDV strains have mono or dibasic residues at their F protein cleavage sites (G/E-K/R-Q-G/E-R↓L) that is cleaved by trypsin-like extracellular proteases, whereas virulent NDV strains contain a polybasic cleavage site (R/K-R-Q-R/K-R↓F) that is cleaved by intracellular protease furin [2,6].

All NDV strains belong to a single serotype, but there is antigenic and genetic diversity among them. Based on phylogenetic analysis of the F gene, NDV strains have been classified into at least 18 genotypes [7,8]. The currently used live-attenuated NDV vaccines are of genotype II, while viruses belonging to genotypes VII and VIII are main cause of outbreaks in Asia and Africa [9–11]. The current vaccines do not completely prevent virulent virus infection and subsequent shedding [12–14]. Several studies have shown that genotype-matched vaccines provide better protection and significant reduction in virus shedding compared to non-genotype-matched vaccines [15–17]. Therefore, there is a need to develop a safe and effective live-attenuated genotype VII NDV vaccine. Although, it is now possible through reverse genetic technology to engineer genotype-matched vaccines, by modifying the F protein cleavage site sequence (FPCS), it is not known which avirulent FPCS is best to generate a genetically stable, safe and effective live-attenuated genotype VII vaccine.

We have previously constructed a genotype VII NDV strain Banjarmasin/010 (Banj) in which the virulent FPCS was modified to avirulent FPCS of NDV strain LaSota [16]. The mutant rBanj-LaSota virus was completely avirulent and induced higher neuralization antibody titer against genotype VII viruses than the commercial B1 or LaSota vaccine. Furthermore, the rBanj-LaSota virus significantly reduced challenge virus shedding from vaccinated birds compared to B1 vaccine [16]. However, the rBanj-LaSota virus did not produce syncytia or plaques in presence of trypsin or chicken embryo allantoic fluid in cell culture. Since syncytia formation plays an important role in spread of paramyxovirus infection [18], the question remained if replacement of FPCS from another APMV serotype would generate a live-attenuated genotype VII virus that is capable of producing syncytia in presence of trypsin-like proteases and hence would be more immunogenic.

We recently evaluated the FPCS of genotype V NDV strain Mexico/01/10 [19]. The FPCS of parental virus was individually mutated to those of avirulent NDV strain LaSota and other APMV serotypes. These mutations affected cell-to-cell fusion activity *in vitro* and the efficiency of F protein cleavage and made the mutant viruses avirulent to chickens. Among the mutant viruses, the recombinant virus containing the FPCS of APMV-2 induced the highest neutralizing antibody titer and completely protected chickens from challenge virus shedding. However, it is not known whether the FPCS of APMV-2 is the most efficient avirulent cleavage site sequence only for genotype V or for all genotypes of NDV.

The aim of this study was to further evaluate the FPCS of a NDV genotype VII virus by replacing the FPCS with the corresponding sites of avirulent NDV strain LaSota and APMV-2, -7 and -8. We wanted to identify an avirulent FPCS that is genetically stable and makes the genotype VII virus more immunogenic. In addition, a rapid neutralization assay was developed using recombinant NDV LaSota (rLaSota) and rBanj-LaSota, both expressing enhanced green fluorescent protein (eGFP), rLaSota-eGFP and rBanj-LaSota-eGFP, respectively, to evaluate the neutralizing antibody titer in the vaccinated chickens. These results will be useful for the development of safe and effective genotype-matched NDV vaccines.

## Material and methods

### Viruses and cells

Chicken embryo fibroblast (DF-1) cell line and human epidermoid carcinoma (HEp-2) cell line were obtained from American Type Culture Collection (ATCC, VA, USA) and grown in Dulbecco’s minimal essential medium (DMEM) with 10% fetal bovine serum (FBS). The modified vaccinia virus strain Ankara (MVA) expressing T7 RNA polymerase was kindly provided by Dr. Bernard Moss (NIH, MD, USA). The highly virulent NDV strain Banj that belong to genotype VII was isolated in Indonesia in 2010 and a reverse genetic system for Banj was previously established [16]. The velogenic NDV strain GB Texas was obtained from USDA (Ames, IA, USA). The recombinant NDVs were grown in 9 to 11-day-old embryonated specific-pathogen-free (SPF) chicken eggs and all virulent virus-related studies were performed in our USDA approved enhanced biosafety level 3 (BSL-3+) facility following the guidelines and approval of IACUC, University of Maryland.

### Plasmid construction and rescue of recombinant viruses

**Fig 1:**
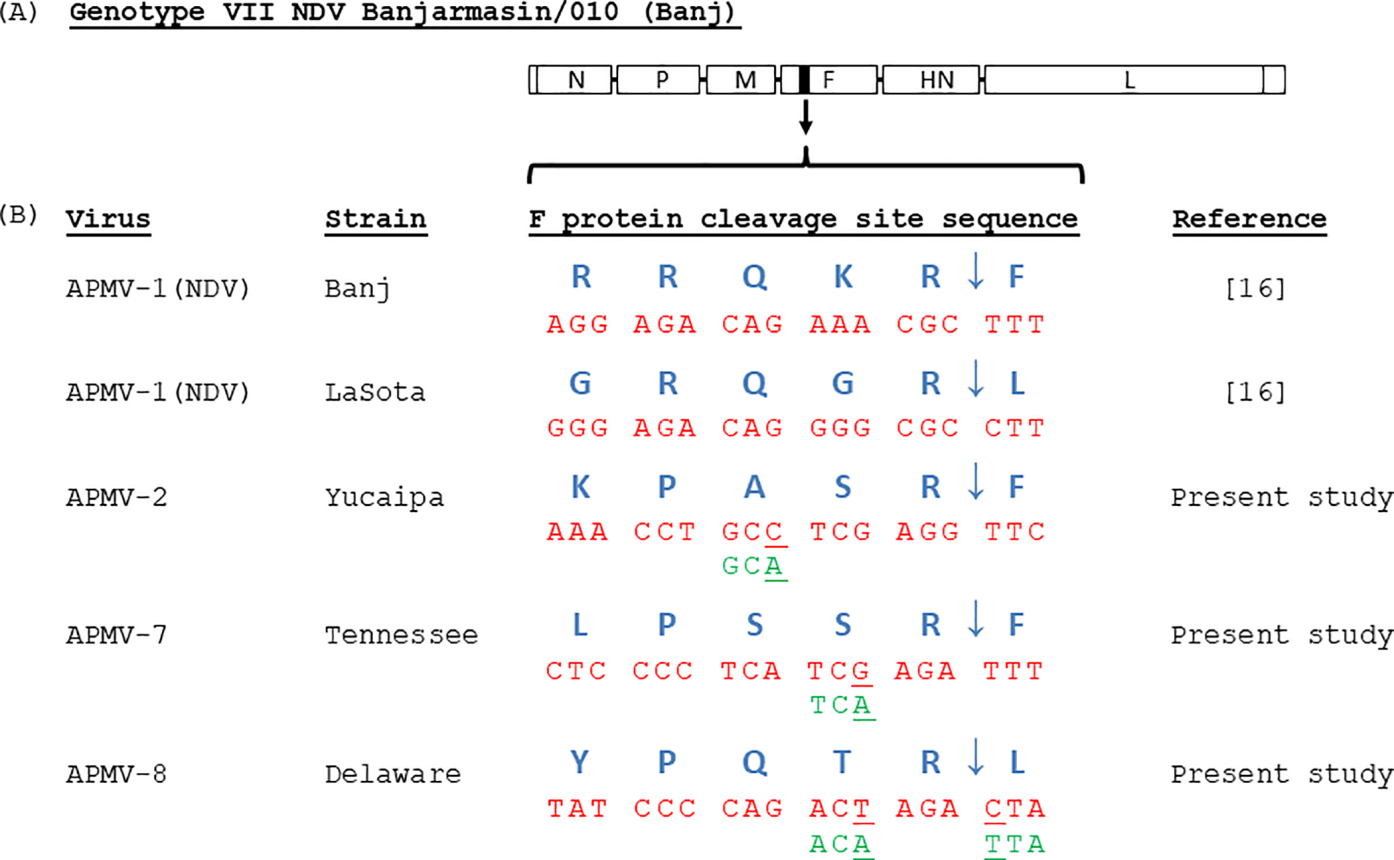
**Gene map of a full-length antigenomic cDNA (A) of genotype VII NDV strain Banjarmasin/010 (Banj) with fusion protein cleavage site (FPCS) modifications (B).** The blue letters indicate amino acid sequences at the FPCS. The arrow indicates the amino acid location where cleavage occurs. The red letters indicate coding nucleotide sequences corresponding to amino acids. The green codon sequences are the original naturally-occurring sequences and the underlined bases indicate the modifications that are introduced in the FPCS to prevent a single nucleotide change generating a basic amino acid residue in the FPCS.

The construction of plasmid pNDV carrying the full length antigenome cDNA of the NDV strain Banj (pBanj) has been described previously [16]. We used overlapping PCR to introduce individual amino acid substitutions into the F gene of NDV strain Banj. The following primer sequences were used for first overlapping fragment: AsiSI-F, 5’-**GCGATCGC**TTATAGTTAG CTCAC; APMV2-R, 5’-GAACCTCGAGGCAGGTTTTCCTCCGGACGTGGCCACCG; APMV7-R, 5’-AAATCTCGATGAGGGGAGTCCTCCGGACGTGGCCACCG; APMV-8-R, 5’-TAGTCTAGTCTGGGGATATCCTCCGGACGTGGCCACCG. The following primer sequences were used for second overlapping fragment: APMV-2-F, 5’-AAACCTGCCTCGAG GTTCATAGGTGCCGTTATTGGCAGTG; APMV-7-F, 5’-CTCCCCTCATCGAGATTTATA GGTGCCGTTATTGGCAGTG; APMV-8-F, 5’-TATCCCCAGACTAGACTAATAGGTGCC GTTATTGGCAGTG; AgeI-R, 5’-**ACCGGT**AGTTTTTTCTTAAGTC. In the primer sequences, restriction enzyme (RE) site sequences are given in bold and the fusion protein cleavage site regions are underlined. Briefly, two overlapping fragments were amplified separately using pBanj as template using LA Taq PCR. The DNA bands were excised, and gel cleaned using NucleoSpin^®^ Gel and PCR Clean-up kit (Clontech, California, USA) following manufacturer’s protocol. The first and second fragments of the PCR were subjected to overlap PCR to amplify the final F gene cassettes incorporating intended cleavage site sequence modifications. The gel-cleaned modified F gene cassettes were cloned into pGEM^®^-T Easy cloning vector (Promega, Wisconsin, USA) and sequenced using gene specific primers. The confirmed FPCS mutant cassettes were subcloned into the pBanj using RE sites AsiSI and AgeI (Fig 1). The sequence of the inserted cassette was confirmed with gene specific primers. These full length pBanj clones were transfected into HEp-2 cells, and rBanj FPCS mutant viruses were recovered as previously described [20]. rBanj FPCS mutant viruses were plaque purified and their F gene cassettes were sequenced to confirm the mutations.

### Intracerebral pathogenicity index test

The pathogenicity of rBanj FPCS mutant viruses was determined by the intracerebral pathogenicity index (ICPI) test in our USDA approved enhanced BSL3 facility in 1-day-old specific pathogen free (SPF) chicks, obtained from Charles River Laboratories, Wilmington, MA, USA. Briefly, for the ICPI test, 0.05 mL of a 1:10 dilution of fresh egg-grown virus was inoculated into group of ten 1-day-old SPF chicks via intracerebral route. At each observation, the birds were scored 0 if normal; 1 if sick; and 2 if dead. The ICPI is the mean score per bird per observation over the 8-day period. Highly virulent viruses give values approaching 2 and avirulent or lentogenic strains give values close to 0 [21]. All the animals used in this study were housed in isolator cages and cared for in accordance with established guidelines, and the experimental procedures were performed with approval from Institutional Animal Care and Use Committee of the University of Maryland.

### Genetic stability of rBani FPCS mutant viruses

The genetic stability of rBanj-LaSota, rBanj-APMV2, rBanj-APMV7 and rBanj-APMV8 was confirmed by passaging the viruses at least 10 times in 9-day-old embryonated chicken eggs and five times in the respiratory tract of 1-day-old chicks. For egg passage, diluted virus in PBS containing 100 fifty-percent-tissue-culture-infectious-dose (TCID_50_) was injected into the allantoic cavity of three 9-day-old embryonated SPF chicken eggs. Three days after incubation at 37°C, the passaged virus was harvested from the allantoic fluid and further passaged in new set of three eggs. For passage in the respiratory tract of 1-day-old chicks, three chicks per virus were inoculated with 100 μL of a 1x10^5^ TCID_50_ of virus by oculo-nasal route. Three days after inoculation, chicks were euthanized; the trachea and lungs were collected and placed in DMEM containing 10X antibiotics (Invitrogen, CA, USA), homogenized and clarified by centrifugation at 2,500 rpm for 15 min. The supernatants were directly inoculated via oculo-nasal route into a new batch of three 1-day-old chicks. To confirm the presence of the virus in the tissue homogenate, 200 μL of the clarified supernatant was injected into the allantoic cavity of 9-day-old embryonated chicken eggs and tested by HA assay. From each passage, total RNA was extracted from infective allantoic fluid of 9-day-old SPF chicken embryos, using TRIzol reagent (Invitrogen, CA, USA). RT-PCR was performed using the Thermoscript RT-PCR kit (Invitrogen, CA, USA) with specific forward and reverse primers to amplify the F gene. The amplified PCR fragments were then sequenced using the BigDye Terminator v3.1 cycle sequencing kit (Applied Biosystems, Texas, USA) in an ABI 3130*xl* genetic analyzer to confirm the presence of the introduced mutations in the passaged viruses.

### Replication of rBanj FPCS mutant viruses in 1-day-old SPF chicks

The replication of rBanj-LaSota, rBanj-APMV2, rBanj-APMV7 and rBanj-APMV8 was compared to rLaSota in 1-day-old chicks. Three 1-day-old chicks per virus group were inoculated with rBanj-LaSota, rBanj-APMV2, rBanj-APMV7, rBanj-APMV8 and rLaSota by one drop in each eye and nostril (100 μL/bird) containing a titer of 1x10^5^ TCID_50_. All the birds were sacrificed at 3 dpi and tissue samples of brain, lung, trachea and spleen were collected. The tissue samples were weighed and homogenized in media containing 10X antibiotics. The supernatants were assayed in DF-1 cells by TCID_50_ method [16].

### Growth kinetics in DF-1 cells and in 9-day old embryonated SPF chicken eggs

NDV titers in PFU/mL were determined by plaque assay in DF-1 cells as previous described [4,16,22]. Briefly, confluent monolayers of DF-1 cells were infected with 10-fold dilution of the respective viruses in DMEM and incubated for 1 h at 37°C. The cells were washed with sterile PBS three times and overlaid with 0.8% methylcellulose in DMEM containing 2% FBS and with or without 10% fresh chicken egg allantoic fluid. At 7 dpi, the cells were fixed with 100% methanol and stained with 1% crystal violet for observation of plaques. NDV titers in TCID_50_ units were determined as previously described [22]. Briefly, confluent monolayers of DF-1 cells were infected with 10-fold dilution of the respective viruses in DMEM and incubated for 1 h at 37°C. The infected cells were maintained in DMEM containing 2% FBS and 10% fresh chicken egg allantoic fluid. At 3 dpi, the cells were fixed with methanol and infected cells were visualized by immunostaining using NDV N protein specific peptide antiserum raised in rabbits, followed by a horseradish peroxidase (HRP) tagged secondary antibody and detection by substrate AEC plus chromogen (Dako, USA). The TCID_50_ titers were calculated by the method of Reed and Muench [23].

To study the *in vitro* growth characteristics of rescued viruses, DF-1 cells grown in six-well plates were infected with each mutant virus, in duplicates at an MOI of 0.001. After 1 h of adsorption, the cells were washed with PBS and overlaid with DMEM containing 2% FBS and 10% fresh allantoic fluid at 37°C. A 200 μL of supernatant medium was collected and replaced with an equal volume of fresh medium every 8 h intervals until 64 h post infection (hpi). Virus yields were quantified in DF-1 cells by TCID_50_ method [22]. To study the replication in 9-day-old embryonated eggs, 200 μL of 100 TCID_50_ virus diluted in PBS was injected into the allantoic cavity of ten 9-day-old embryonated SPF chicken eggs. Three days after incubation at 37°C, the viruses were harvested from the allantoic fluid and titrated by hemagglutination (HA) assay [21]. The ability of the rBanj FPCS mutant viruses to induce CPE and form plaques was characterized by infecting DF-1 cells with rLaSota or rBanj FPCS mutant viruses in the presence or absence of 10% fresh chicken embryo allantoic fluid. Cleavage efficiency of the F proteins of vaccine viruses was evaluated by Western blot analysis with anti-NDV F rabbit polyclonal antiserum [16].

### Immunization and challenge experiments in SPF chicks

The immunization studies were done in our BSL-2 facility and challenge studies were performed at our USDA approved enhanced BSL-3 facility. One-day-old SPF chicks were randomly assigned to 6 treatment groups of 20 birds. All birds were housed in separate poultry isolation chambers with *ad libitum* access to feed and water. The birds were vaccinated via oculo-nasal route with 100 μL of 1x10^5^ TCID_50_ of rLaSota, rBanj-LaSota, rBanj-APMV2, rBanj-APMV7 and rBanj-APMV8. For the control group, 100 μL of sterile PBS was administered. Blood samples were collected at 1, 2, and 3 weeks post-immunization (WPI) for analyzing NDV antibody levels by hemagglutination inhibition (HI) and virus neutralization assays. At 3 WPI, 10 birds in each group were challenged with virulent NDV strain Banj and the remaining 10 birds were challenged with virulent NDV strain Texas GB using a titer of 100 chicken lethal dose 50 (CLD_50_) per bird by oculo-nasal route. All birds were observed daily for clinical signs (death, paralysis, and torticollis) until 10 days post challenge. In order to determine shedding of the vaccine and challenge viruses, oral and cloacal swabs were collected on day 4 post vaccination and on day 4 post challenge from all chickens.

### Development of a reliable and rapid virus neutralization assay

A reliable and rapid virus neutralization assay is necessary to determine the immune status of the vaccinated chickens. Traditionally, plaque reduction neutralization tests and inhibition of the cytopathogenic effects are used for NDV neutralization assay. The conventional assays are time consuming and needs expertise in performing the assay. In this study, an alternative convenient neutralization assay for measuring NDV neutralizing antibody level in serum samples was developed. Briefly, recombinant NDV (rNDV) expressing stable eGFP were generated by inserting the eGFP gene cassette between P and M genes, namely, rLasota-eGFP and rBanj-LaSota-eGFP, using reverse genetic technology. The neutralization activity was measured by the suppression of fluorescence of rLaSota-eGFP and of rBanj-LaSota-eGFP by the test serum. Briefly, two-fold serial dilutions of complement inactivated chicken serum samples were made in a 96-well plate and incubated with 1x10^3^ TCID_50_ of rNDV (rLasota-eGFP and rBanj-LaSota-eGFP) at 37 °C for 1 h. The mixture was then transferred to DF-1 cells in a 96-well plate, incubated for 2 h, and replaced with DMEM containing 2% FBS and 10% fresh allantoic fluid. After incubation at 37°C for 48 h, the cells were washed with PBS and fixed with 4% paraformaldehyde. The experiment was done in two replicates. The fluorescence intensity was measured using a microplate Reader (Tecan, Infinite® M1000) with fluorescence intensity reading (Excitation wavelength: 490 nm, Excitation bandwidth: 5 nm, Emission wavelength: 525 nm, Emission bandwidth: 10 nm). The neutralization titer was defined as the reciprocal of the highest serum dilution that resulted in 50% reduction of mean eGFP fluorescence. The neutralization titers obtained using the rNDV-eGFP reporter assay was validated using HI assay.

## Results

### Construction and recovery of rBanj FPCS mutant viruses

In the present study, three FPCS mutant cDNAs of NDV strain Banj [16] containing FPCS of APMV2, APMV7 and APMV8 were constructed (Fig 1). The mutant viruses were recovered by procedures described previously [20]. The recovered viruses were designated as rBanj-APMV2, rBanj-APMV7 and rBanj-APMV8, respectively. The nucleotide sequences of the complete F gene were confirmed for the presence of introduced mutation and also for the lack of adventitious mutations.

### Pathogenicity of rBanj FPCS mutant viruses in 1-day-old SPF chicks

The pathogenicity of rBanj FPCS mutant viruses rBanj-LaSota, rBanj-APMV2, rBanj-APMV7 and rBanj-APMV8 were evaluated by ICPI test in 1-day-old SPF chicks [21]. The ICPI values of rBanj-LaSota, rBanj-APMV2, rBanj-APMV8 and rLaSota were 0.00 and the ICPI value of rBanj-APMV7 was 0.25 (Table 1). These results suggest that the rBanj FPCS mutant viruses are avirulent to chickens and have the potential to be used as live-attenuated vaccines for ND.

**Table 1:** Pathogenicity of rBanj FPCS mutant viruses in 1-day-old SPF chicks.

Cleavage site mutant virus	ICPI score
**rBanj-LaSota**	0.00
**rBanj-APMV2**	0.00
**rBanj-APMV7**	0.25
**rBanj-APMV8**	0.00
**rLaSota**	0.00

### Genetic stability of the rBanj FPCS mutant viruses

**Fig 2:**
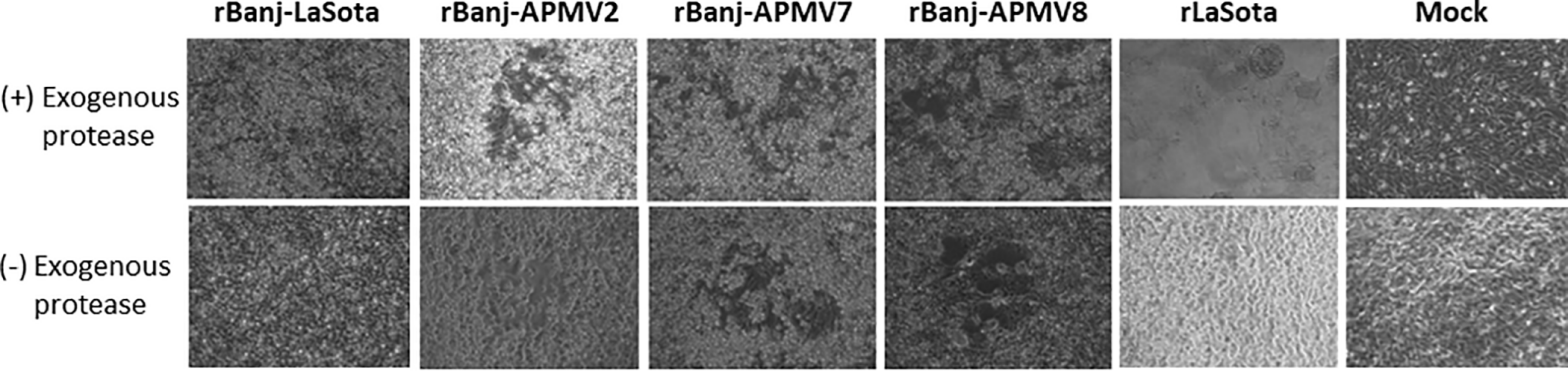
Cytopathogenicity of rBanj FPCS mutants in DF-1 cells. The cells were infected at an MOI of 1 with each of the recombinant viruses. After 3 days, the cytopathic effects (CPE) of each virus infected monolayer was examined under microscope (10X). Ten percent fresh chicken embryo allantoic fluid was used as the source of exogenous protease.

**Fig 3:**
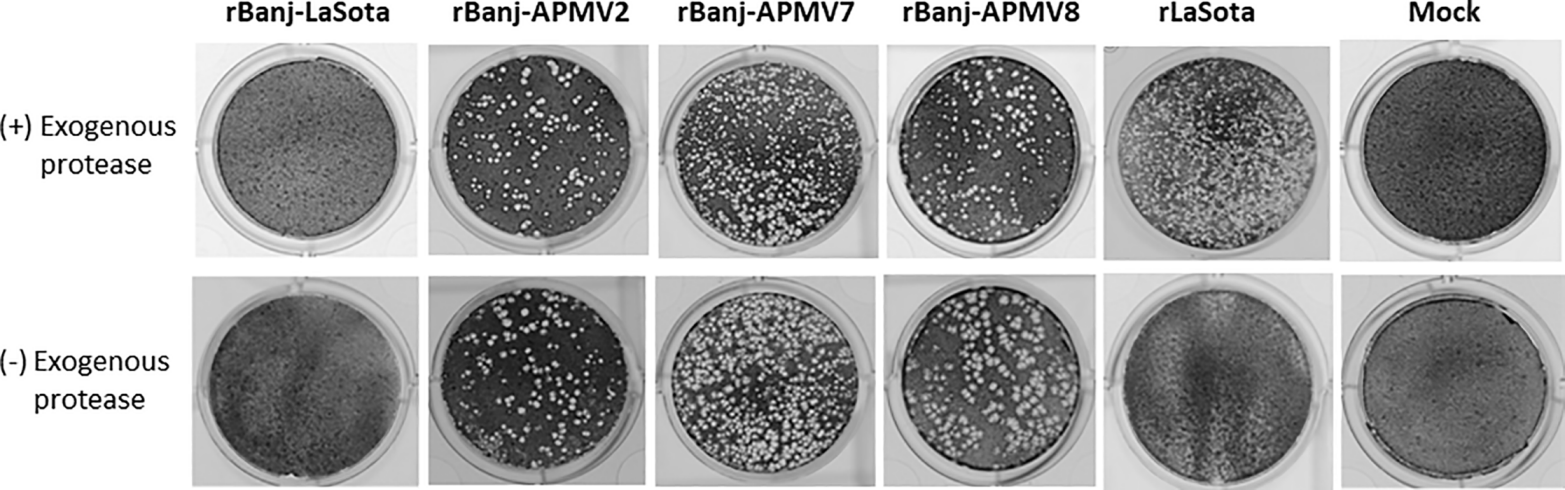
Plaque morphology of rBanj FPCS mutants in DF-1 cells. Confluent monolayer of DF-1 cells were infected with each of the recombinant viruses. The infected cells were overlaid with 0.8% methyl cellulose in DMEM, 2% FBS, with or without 10% fresh chicken embryo allantoic fluid as a source of exogenous protease. The viral plaques were obtained after staining with 1% crystal violet.

The genetic stability of rBanj FPCS mutant viruses was evaluated in 9-day-old embryonated SPF chicken eggs and in 1-day-old SPF chicks. Ten serial passages in embryonated eggs and five passages in the respiratory tract of 1-day-old chicks did not show changes in the nucleotide sequence of FPCS, indicating a lack of reversion or introduction of adventitious mutations. Sequence analysis of 10 individual plaques of each passaged virus did not show any nucleotide changes, conforming that all the rBanj FPCS mutant viruses were genetically stable. (Figs 2 and 3)

### Fusion protein cleavage

**Fig 4:**
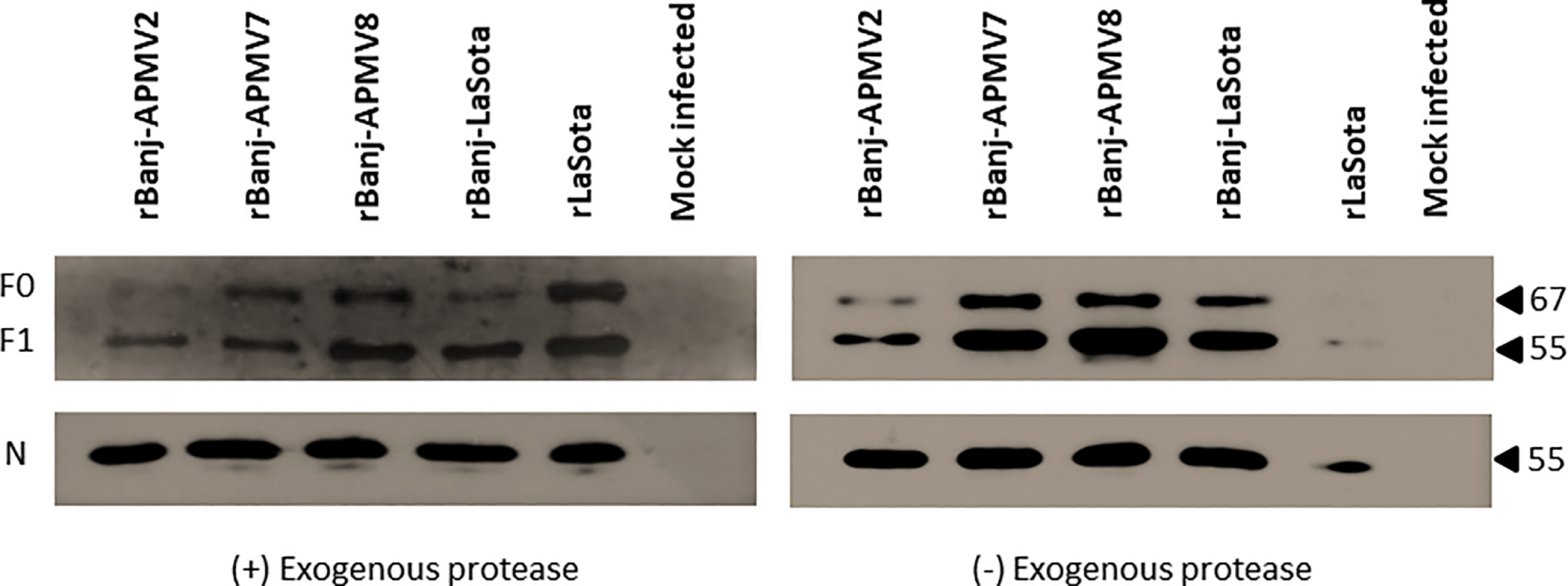
F protein cleavage of rBanj FPCS mutants and rLaSota in DF-1 cells. The cells were infected with respective viruses at an MOI of 1. The cell lysates were collected at 24 h post infection. Western blot was performed using a NDV F cytoplasmic tail anti-peptide rabbit serum. Ten percent fresh chicken embryo allantoic fluid was used as the source of exogenous protease.

The cleavage of the F protein of rBanj FPCS mutant viruses was determined in the presence and absence of exogenous protease (10% fresh chicken allantoic fluid) by Western blot analysis using NDV F cytoplasmic tail anti-peptide rabbit serum (Fig 4). The results showed that the F proteins of rBanj-LaSota, rBanj-APMV2, rBanj-APMV7 and rBan-APMV8 were cleaved either in the presence or the absence of exogenous protease (10% fresh chicken allantoic fluid) after 24 h infection in DF-1 cells, whereas the F protein of strain LaSota was cleaved only in the presence of exogenous protease (10% fresh chicken allantoic fluid) (Fig 2 and Fig 3).

### Biological characterization of rBanj FPCS mutant viruses

**Fig 5:**
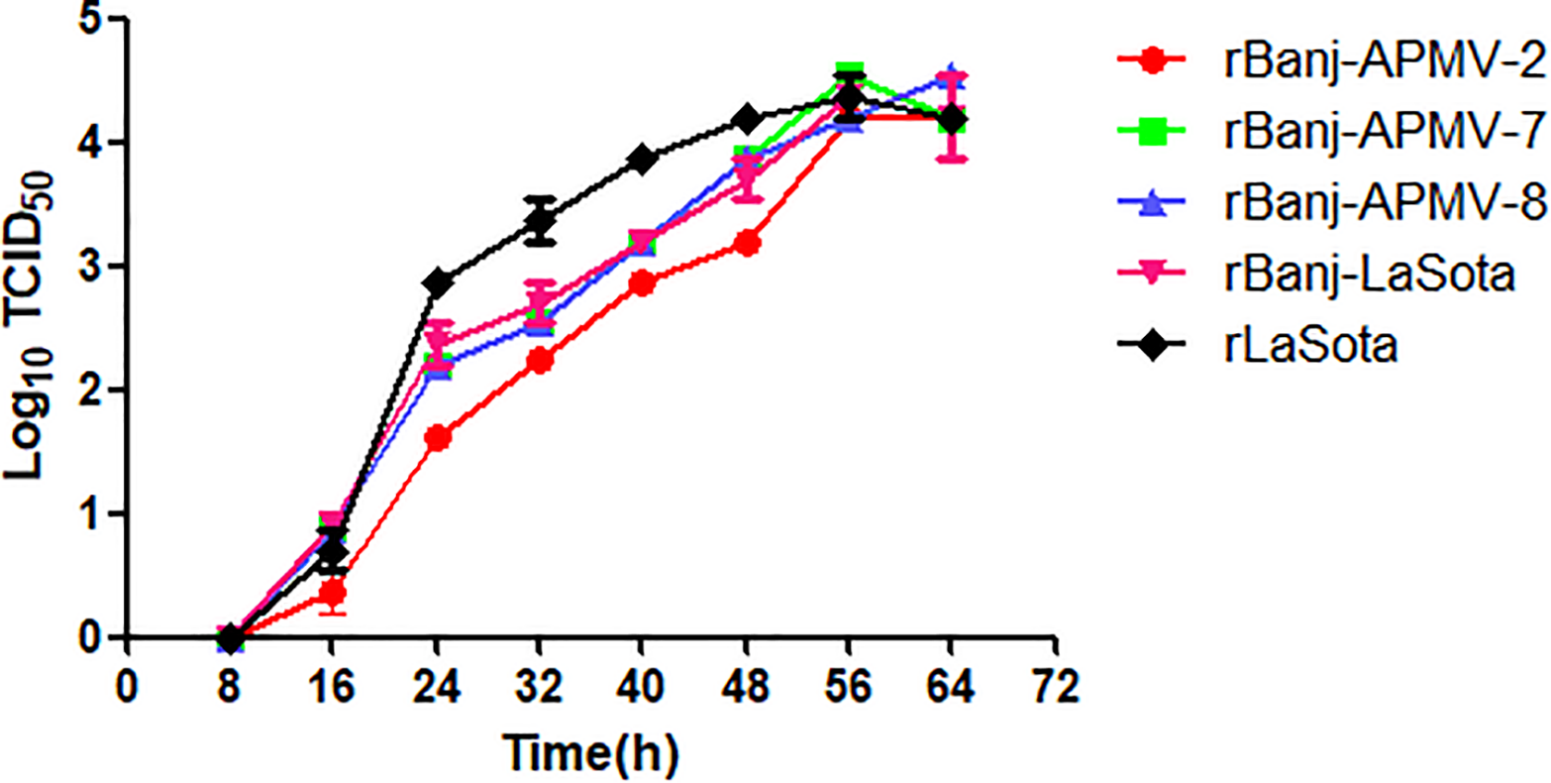
Growth kinetics of rLaSota and rBanj FPCS mutant viruses in DF-1 cells. Cells were infected at an MOI of 0.001 of each virus and the cell culture supernatant was collected at 8 h intervals for 64 h. All virus titers are expressed as mean log_10_ TCID_50_/mL ± SEM (standard error of the mean).

**Fig 6:**
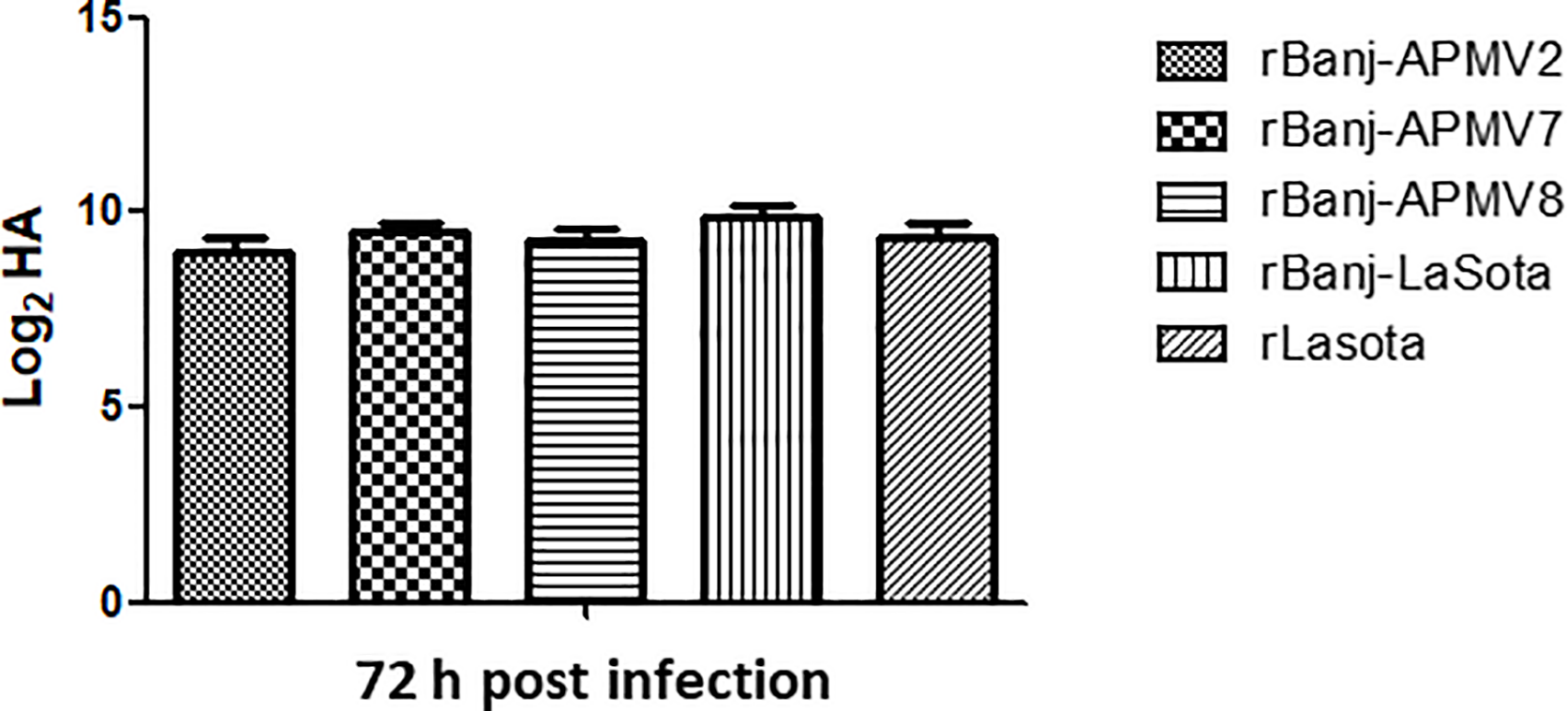
Growth kinetics of rLaSota and rBanj FPCS mutant viruses in 9-day-old embryonated SPF chicken eggs. Two hundred μL of 100 TCID_50_ of each virus in PBS was injected into the allantoic cavity of ten 9-day-old embryonated SPF chicken eggs. Three days after incubation at 37°C, the allantoic fluids was harvested and titrated by hemagglutination (HA) assay. The bars are the means of HA titers. Error bars indicate standard error of the mean.

Plaque assay in DF-1 cells showed that rBanj-APMV2, rBanj-APMV7 and rBanj-APMV8 produced plaques similar to rLaSota (Fig 3), whereas rBanj-LaSota produced only single cell infection and no plaques [16]. The multi-cycle growth kinetics of the rBanj FPCS mutant viruses were determined in DF-1 cells and viral titers were analyzed by the TCID_50_ assay (Fig 5). The FPCS mutant viruses replicated exponentially until 56 hpi, after which they reached a plateau. The replication of rBanj-APMV2 was slightly lower than other FPCS mutant viruses but reached similar viral titer at 56 hpi. In 9-day old embryonated eggs, all the viruses reached similar HA titers at 3 dpi at 37°C (Fig 6).

### Replication and tissue tropism of rBanj FPCS mutant viruses in one-day-old SPF chicks

**Fig 7:**
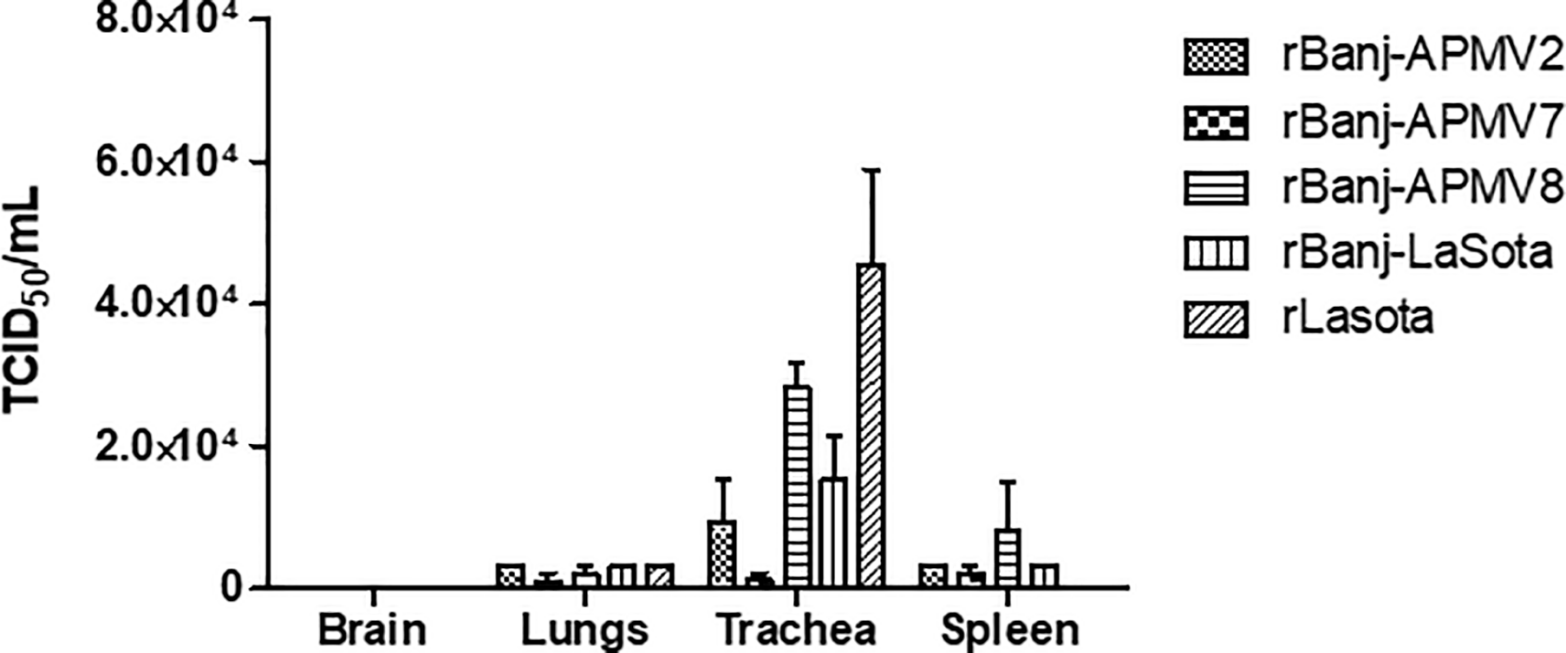
Virus titers and tissue tropism of rBanj FPCS mutant viruses in 1-day-old chicks following oculo-nasal inoculation. Tissue samples from brain, lungs, trachea, and spleen of 3 chickens (n  =  3) from each indicated virus group were collected on day 3 post infection, and virus titers were determined by TCID_50_ assay. The mean virus titers for each tissue sample from 3 chickens are shown. Error bars indicate standard error of the mean.

The replication and tissue tropism of rBanj FPCS mutant viruses were evaluated in 1-day-old SPF chicks. Chicks in groups of 3 were inoculated with rBanj-LaSota, rBanj-APMV2, rBanj-APMV7, rBanj-APMV8 and rLaSota by one drop in each eye and nostril (100 μL/bird) with a titer of 1x10^5^ TCID_50_. All the birds were sacrificed at 3 dpi and tissue samples from brain, lungs, trachea and spleen were collected. The viruses were detected in all organs except brain. Comparison of the viral titers of the tissue samples showed that all mutant viruses replicated well in trachea except rBanj-APMV7, whereas in spleen and lungs all the viruses replicated to similar levels (Fig 7).

### Evaluation of immunogenicity and protective efficacy of rBanj FPCS mutant viruses in SPF chickens

**Fig 8:**
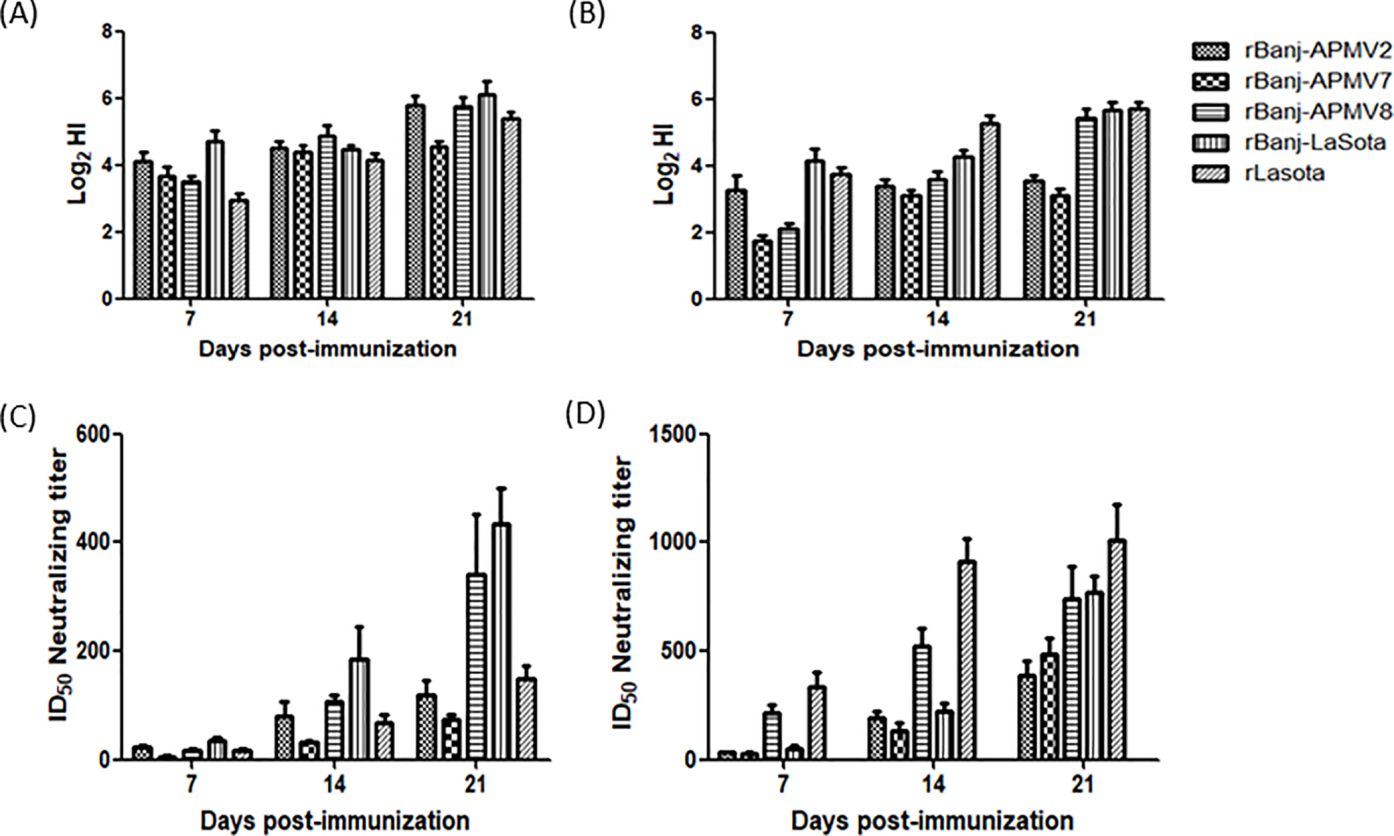
**Induction of NDV-specific serum antibodies in chickens in response to vaccination with rBanj FPCS mutant viruses, determined by HI (A and B) and serum neutralization assays (C and D).** (A and B) Twenty 1-day-old chicks per group were inoculated with 100 μL of (1x10^5^ PFU) virus via oculo-nasal route. Serum samples were collected at 1, 2, and 3 weeks post inoculation. NDV-specific antibodies were determined by hemagglutination inhibition (HI) assay sing 4 HA units of rBanj-LaSota (A) and rLaSota (B). (C and D) The serum neutralizing titers of the vaccinated birds against rBanj-LaSota-eGFP (C) and rLaSota-eGFP (D) viruses are shown. Two-fold serial dilutions of complement-inactivated serum samples were made in a 96-well plate and incubated with 1x10^3^ TCID_50_ of the recombinant virus at 37°C for 1 h. After incubation at 37°C for 48 h, the cells were washed with PBS and fixed using 4% paraformaldehyde. The fluorescence intensity was measured using a micro plate reader (Tecan, Infinite® M1000) at 490 nm. The neutralization titer was defined as the reciprocal of the highest serum dilution that resulted in 50% reduction in mean eGFP fluorescence.

The immunogenicity of the rBanj FPCS mutant viruses was evaluated in 1-day-old SPF chicks. Groups of 20 birds were inoculated with 100 μL of 10^5^ TCID_50_ of rBanj-LaSota, rBanj-APMV2, rBanj-APMV7, rBanj-APMV8, rLaSota or PBS. None of the chicks infected with rLaSota or rBanj FPCS mutant viruses showed clinical signs, suggesting that all viruses were avirulent to chickens. All infected birds were seropositive for antibodies against NDV by HI test. The differences between the HI titers to rBanj-LaSota and rLaSota in birds vaccinated with rLaSota and rBanj-LaSota are unexpectedly minimal (Fig 8A and 8B).

**Fig 9:**
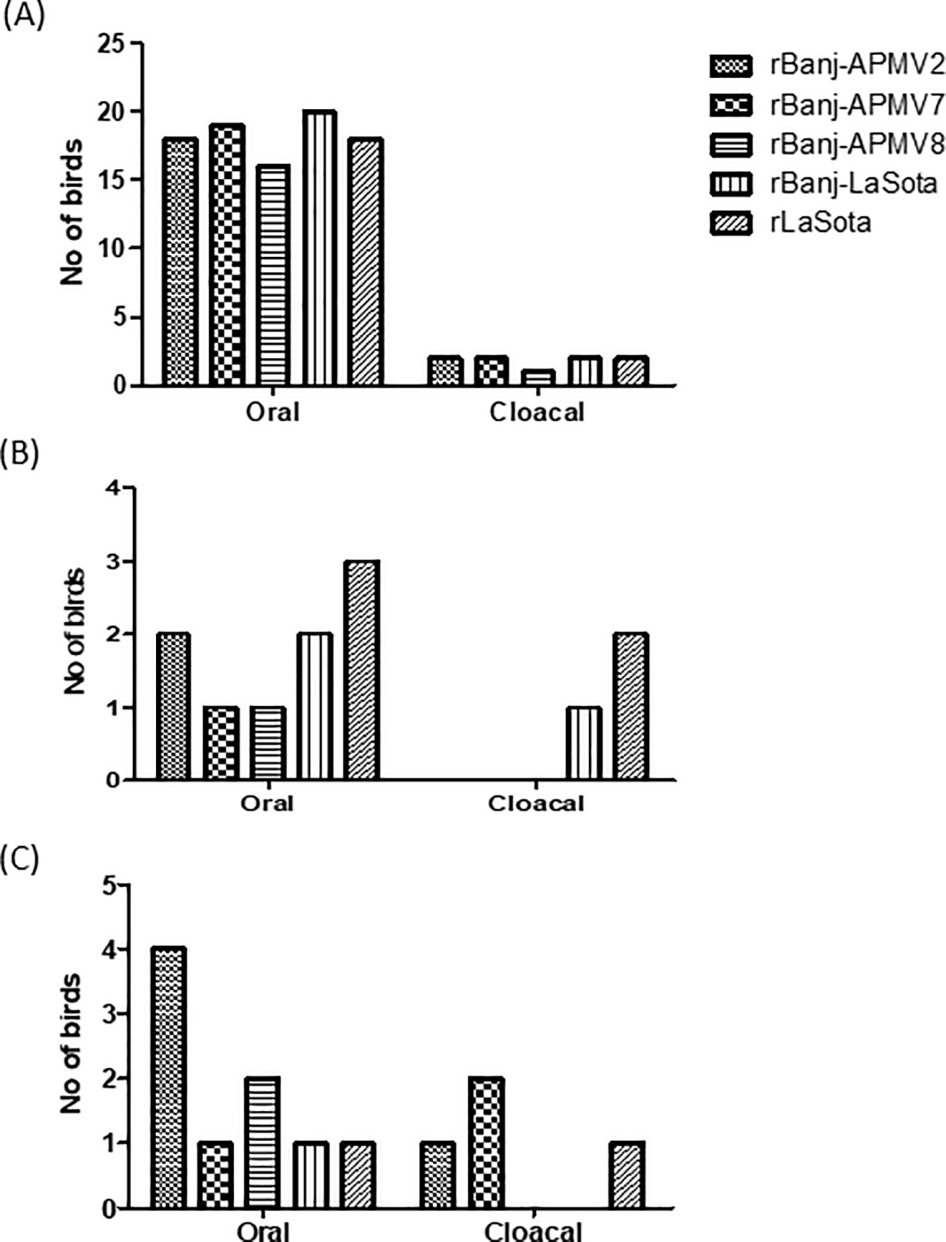
**Replication and shedding of vaccine viruses (A) and challenge viruses (B and C) in birds vaccinated at 1-day-old and challenged at 3-week-old, respectively.** (A) Chicks in groups of 20 were infected with rBanj FPCS mutants via oculo-nasal route. At day 4 post inoculation (PI), oral and cloacal swabs were collected and analyzed for the presence of virus by inoculation into 9-day-old embryonated SPF chicken eggs. 4 dpi, the allantoic fluid was tested by hemagglutination (HA) assay for the presence of the virus. Shedding of virulent Banjarmasin (Banj) (B) and GB Texas (C) challenge viruses from oral and cloacal swabs. Groups of 10 birds per virus vaccinated with rBanj FPCS mutant viruses at 1-day-old and challenged by the oculo-nasal route with 100 CLD_50_ virus in 200 μL volume. Oral and cloacal swabs were collected on day 4 post challenge and samples were assayed for virus by inoculation into 9-day-old embryonated chicken eggs and subsequently tested for virus presence by hemagglutination (HA) assay.

Birds from all the groups were challenged at 3 WPI via oculo-nasal route with virulent NDV strains Texas GB (belonging to genotype II) and Banj (belonging to genotype VII) at 100 CLD_50_ (200 μL of 10^4^ TCID_50_) per bird. Our results showed that all the birds immunized with rLaSota or rBanj FPCS mutant viruses were completely protected from virulent Texas GB and virulent Banj challenges without any apparent clinical sign. In contrast, all the birds in the PBS control group had to be euthanized on day 4 or 5 post challenge based on clinical scores. In order to determine shedding of the vaccine and challenge viruses, oral and cloacal swabs were collected on day 4 post vaccination and on day 4 post challenge from all the chickens. The vaccine virus shedding results showed the presence of virus from most of the oral swabs and a few of the cloacal swabs (Fig 9A). The challenge virus shedding results showed that neither of the vaccine gave 100% protection from virulent virus shedding (Fig 9B and 9C). But there was at least 50% reduction in challenge virus shedding when the vaccine virus and the challenge virus were from the same genotype and higher shedding was observed when the challenge virus was from a different genotype. Serum samples collected at 7, 14 and 21 dpi were analyzed by HI and virus neutralization assays against the two viruses, rBanj-LaSota and rLaSota. The serum from rLaSota immunized 1-day-old chicks showed higher HI titer to rLaSota on day 14, compared to the titers of rBanj-LaSota mutant viruses. The serum from rBanj-LaSota immunized chicks showed higher HI titers to rBanj-LaSota on day 7, but lower HI titer to rLaSota (Fig 8A and 8B).

The neutralization assay using rNDV-eGFP showed that all the serum samples neutralized both the eGFP viruses (rLaSota-eGFP and rBanj-LaSota-eGFP), but the neutralization titers were at least 2-fold higher to the genotype-matching strain than to the non-genotype matching strain (Fig 8C and 8D). The eGFP fluorescence inhibition-based neutralization assay is an alternate convenient neutralization assay for NDV. The results obtained from the serum neutralization and HI assays of the serum samples obtained from 1-day-old immunized chicks showed that the genotype-matched vaccines induced better neutralizing antibody response.

## Discussion

The FPCS sequence of avirulent NDV strain LaSota has been used to generate live-attenuated genotype-matched NDV vaccines [15,16,19,24–28]. However, it is not known if this is the best avirulent FPCS to develop safe and effective genotype-matched NDV vaccines. Therefore, in this study we have compared the avirulent FPCS of APMV-2, APMV-7 and APMV-8 with the avirulent cleavage site of strain LaSota in developing a safe and effective genotype VII NDV vaccine. The FPCS sequences of APMV-2, APMV-7 and APMV-8 were chosen because these sites will require more number of mutations to change back to virulent cleavage site motif. To further stabilize the FPCS, the codon of amino acids at the FPCS of APMV-2, APMV-7 and APMV-8 were changed so that they will require more number of mutations to become basic residues.

In this study, the rBanj FPCS mutant viruses were readily recovered with *in vitro* growth kinetics similar to that of NDV strain LaSota. This result indicated that the amino acid modification at the FPCS of strain Banj did not detectably affect virus recovery and replication. *In vitro* characterization of the mutant viruses showed that rBanj-APMV2, rBanj-APMV7, and rBanj-APMV8 induced syncytia and plaque formation in the presence or absence of exogenous protease; whereas, rBanj-LaSota induced single-cell infection and did not form plaques as observed previously [16]. Analysis of the F protein showed that it was efficiently cleaved in all the mutant viruses in presence or absence of exogenous protease. The F protein of rLaSota was only cleaved in the presence of exogenous protease. Our results differ from those of Kim et al. (2017), in which genotype V NDV strain Mexico/01/10 containing APMV-2, -7 and -8 FPCS did not produce syncytia or plaques in presence or absence of protease. These results showed that efficient cleavage of the F protein is a prerequisite for syncytia formation. But the cleavage may not necessarily produce a biologically active molecule, suggesting that FPCS also determines whether the cleavage results in a conformation that is biologically active.

All rBanj FPCS mutant viruses were highly attenuated, confirming that the amino acid sequence at the FPCS is a major determinant of NDV virulence. A major concern for using genetically modified live-attenuated NDV vaccines is that the mutated F protein cleavage site might revert to wild type sequence after passage in chickens. Therefore, to test the genetic stability, all the mutant viruses were passaged ten times in chicken embryos or five times in the respiratory tract of 1-day-old chicks. We then sequenced the F gene, in each case there was no sequence change, indicating that these mutant viruses were genetically stable. However, larger studies involving commercial chickens are needed to confirm the genetic stability of these recombinant attenuated viruses.

All the rBanj FPCS mutant viruses replicated to similar levels in lungs, trachea and spleen of 1-day-old chicks. The tissue tropism was similar among the mutant viruses and rLaSota. None of the mutant viruses replicated in brain. Surprisingly, rBanj-LaSota, which did not produce syncytia *in vitro*, also replicated efficiently in 1-day-old chicks. Similar results were also found with genotype V FPCS mutant viruses [5]. This result suggests that syncytia formation is not an absolute requirement for NDV replication.

One-day-old chicks vaccinated with rBanj FPCS mutants and rLaSota were fully protected from disease and mortality caused by either genotype-matched (strain Banj) or mis-matched (strain GB Texas) NDV challenge. These results show that the rBanj FPCS mutants and rLaSota viruses are capable of fully protecting chickens from disease and death following infection with virulent viruses of different genotypes. Challenge virus shedding results showed that neither rBanj FPCS mutants nor rLaSota completely prevented virus shedding. However, it was observed that there was comparatively less virus shedding in genotype-matched vaccines than in genotype-mis-matched vaccine. These results support previously reported data by us and others [15–17,26,28,29].

The amino acid sequence identities of the F and HN proteins between the Indonesian strain Banj and the vaccine strain LaSota are 74% and 76%, respectively; compared to 93% and 91% for F and HN protein between the strain GB Texas and LaSota. This suggests that the level of amino acid sequence divergence between the genotype mis-matched vaccine and challenge viruses could be the reason for more virus shedding. Our results further showed that among the rBanj FPCS mutant viruses there was comparatively less shedding of challenge viruses when the 1-day-old chicks were vaccinated with rBanj-LaSota or rBanj-APMV8. One possible explanation could be that the mutant viruses containing the FPCS of LaSota and APMV-8 probably replicated more efficiently *in vivo* than the others. Our results differ from those of Kim et al. (2017), in which the FPCS of APMV-2 was found to be best for generating a live-attenuated genotype V NDV vaccine. These results suggest that the most efficient FPCS can vary from genotype to genotype.

HI antibody titers of 1-day-old chicks vaccinated with the rBanj FPCS mutant viruses and rLaSota were not significantly different when tested against either rBanj-LaSota or rLaSota. Analysis of the 7-day post-immunization serum samples showed slightly higher HI titer against rBanj-LaSota, indicating that this virus probably replicated more efficiently than other rBanj FPCS mutant viruses and rLaSota. Analysis of the 14- and 21-day post-immunization serum samples showed that all rBanj FPCS mutant viruses and rLaSota induced good HI titers, but in general, the HI titers were slightly higher against genotype-matched virus than against genotype-mis-matched virus. This result supports previous observations that HI titers can vary depending on the genotype of the virus used for testing [27].

Although the HI antibody titer correlates well with serum neutralization antibody titer [1], the HI assay detects antibodies specific for HN antigen, while the major protective antigen of NDV is the F protein [5]. Therefore, the serum samples of 1-day-old vaccinated chicks were also tested by a neutralization assay using rBanj-LaSota-eGFP and rLaSota-eGFP. This rNDV-eGFP reporter virus assay provides direct quantification of virus replication based on the levels of eGFP expression. This assay is reliable and rapid compared to conventional NDV neutralization assays, which will be useful in diagnostic and molecular virology. Using this novel assay, we have shown that, in general, the serum neutralizing antibody titers of vaccinated chicks were higher against genotype-matched challenge virus than to genotype-mis-matched challenge virus, which was not performed in previous studies [5,15,19,26,28]. In addition, our results suggest that the HI test may not be a useful assay for quantitative determination of NDV neutralizing antibody titer in serum samples. Furthermore, the replication ability of the mutant viruses in trachea (Fig 7) appears to correlate with immunogenicity (Fig 8).

In conclusion, this study showed that the FPCS of LaSota and APMV-8 are preferred sequences for generating genotype VII NDV vaccines by reverse genetics. Our results demonstrated that the most efficient avirulent FPCS can vary from genotype to genotype. Further, our study confirmed previous findings that a genotype-matched vaccine is superior than a genotype-mis-matched vaccine. This study also suggest that the structure of the cleaved F protein is important for syncytia formation. Formation of biologically active F structure not only requires the sequence of FPCS but also the sequence of F protein. Additionally, we developed a novel and reliable NDV-eGFP reporter virus-based neutralization assay that could be used for rapid screening of neutralizing antibodies against NDV. Our results will contribute to the development of improved genotype-matched vaccines against Newcastle disease.
